# Specialization of a mobile, apex predator affects trophic coupling among adjacent habitats

**DOI:** 10.1038/s41598-021-99017-1

**Published:** 2021-10-04

**Authors:** Carl S. Cloyed, Rachel M. Wilson, Brian C. Balmer, Aleta A. Hohn, Lori H. Schwacke, Eric S. Zolman, Mandy C. Tumlin, Randall S. Wells, Aaron A. Barleycorn, Jason B. Allen, Ruth H. Carmichael

**Affiliations:** 1 Dauphin Island Sea Lab, Dauphin Island, AL 36528 USA; 2 Department of Marine Sciences, University of South Alabama, Mobile, AL 36688 USA; 3 Department of Earth, Ocean, and Atmospheric Science, Florida State University, Tallahassee, FL 32306 USA; 4 National Marine Mammal Foundation, San Diego, CA 92106 USA; 5 NOAA, National Marine Fisheries Service, Southeast Fisheries Science Center, Beaufort, NC 28516 USA; 6 Louisiana Department of Wildlife and Fisheries, Baton Rouge, LA 70808 USA; 7 Chicago Zoological Society’s Sarasota Dolphin Research Program, c/o Mote Marine Laboratory, Sarasota, FL 34236 USA

**Keywords:** Behavioural ecology, Community ecology, Stable isotope analysis

## Abstract

Mobile, apex predators are commonly assumed to stabilize food webs through trophic coupling across spatially distinct habitats. The assumption that trophic coupling is common remains largely untested, despite evidence that individual behaviors might limit trophic coupling. We used stable isotope data from common bottlenose dolphins across the Gulf of Mexico to determine if these apex predators coupled estuarine and adjacent, nearshore marine habitats. δ^13^C values differed among the sites, likely driven by environmental factors that varied at each site, such as freshwater input and seagrass cover. Within most sites, δ^13^C values differed such that dolphins sampled in the upper reaches of embayments had values indicative of estuarine habitats while those sampled outside or in lower reaches of embayments had values indicative of marine habitats. δ^15^N values were more similar among and within sites than δ^13^C values. Data from multiple tissues within individuals corroborated that most dolphins consistently used a narrow range of habitats but fed at similar trophic levels in estuarine and marine habitats. Because these dolphins exhibited individual habitat specialization, they likely do not contribute to trophic coupling between estuarine and adjacent marine habitats at a regional scale, suggesting that not all mobile, apex predators trophically couple adjacent habitats.

## Introduction

Food webs comprise distinct compartments that are often thought to be coupled at top trophic levels by mobile, apex predators^1–3^. Trophic compartments often form in association with distinct habitats, such as rivers, estuaries, nearshore coastal habitats, and offshore pelagic and benthic habitats^1,4^. Landscapes are mosaics of habitats that are, in turn, composed of microhabitats, and associated trophic compartments can form at each of these scales of community organization. Compartmentalization can reduce broad-scale impacts across the whole food web by isolating perturbations and subsequent trophic cascades to those compartments^5,6^. Mobile, apex predators can further stabilize food webs by moving among habitats and microhabitats, connecting energy and nutrients among them, and exerting continual top-down control^7,8^. As examples, many top fish predators are thought to forage across the energetically distinct benthic and pelagic zones in lakes and many sharks and small cetaceans forage in both estuarine and marine habitats along coasts, patterns believed to stabilize those systems as a whole^1,8,9^. This pattern of homogenizing trophic compartments at higher trophic levels is assumed to be widespread and common^3,10^, especially in aquatic habitats^8,11,12^.

Counter to this assumption, mobile, apex predators do not always act in ways that effectively couple habitats^9,13,14^. Many apex predators have broad habitat niches and are considered generalists at the population scale, and as they move through multiple habitats and feed on many prey types, have the potential to connect these associated trophic compartments^15^. But ecologists are becoming increasingly aware that many generalist species are composed of individual specialists that use only a subset of the available habitats and prey^16–19^. Individuals may function as specialists while the population and species may ecologically function as generalists^14,16,19^. Although populations can be composed of both individual specialists and generalists, many tradeoffs in resource acquisition and behavior, like high individual site fidelity, can prevent populations from being a mixed composition of individuals^20–22^. One potent effect of individual specialization is that specialists often focus on a single habitat or trophic compartment and do not effectively overlap adjacent food webs^9,13,14^. For example, individual perch in temperate lakes use only pelagic or littoral habitats and do not couple them as previously thought^13^. Similarly, individual bull sharks in southern Florida show long-term use of habitats along an estuarine-to-marine gradient but do not effectively couple these habitats^9^. The number of apex predators that function as individual specialists suggests that trophic coupling among habitats may not be as common as currently assumed^16,17^. However, few studies have linked individual habitat and diet use to compartmentalized rather than coupled food webs but see^9,13,14^, and it is unknown how widespread compartmentalization is in contrast to coupling.

Common bottlenose dolphins (*Tursiops truncatus truncatus;* hereafter referred to as dolphins) are globally ubiquitous, habitat generalists, and many nearshore and offshore populations have large ranges with extensive movements encompassing multiple habitats^23–25^. Dolphin populations that inhabit estuarine waters often show high site fidelity and individual habitat specialization, even if they are largely dietary generalists within that narrow habitat range^18,26–29^. Although they are highly mobile apex predators, most estuarine dolphins may not functionally couple the trophically distinct estuarine and adjacent marine habitats because their small ranges restrict their habitat use and they infrequently use adjacent marine habitats^26,30^. For example, telemetry, photo-identification, and stable isotope studies have demonstrated structured populations in which dolphins from upper estuarine habitats rarely, if ever, use adjacent nearshore, marine habitats^26,31–34^. Many dolphin populations also have developed specialized foraging behaviors that target certain prey^21,35,36^. As a result, there is evidence that estuarine dolphins may maintain trophic compartments along an estuarine-to-marine habitat gradient^9,37,38^, a feeding pattern that is inconsistent with the widespread belief that trophic coupling is common in top predators that can move among aquatic habitats^8,11,12^.

**Figure 1:**
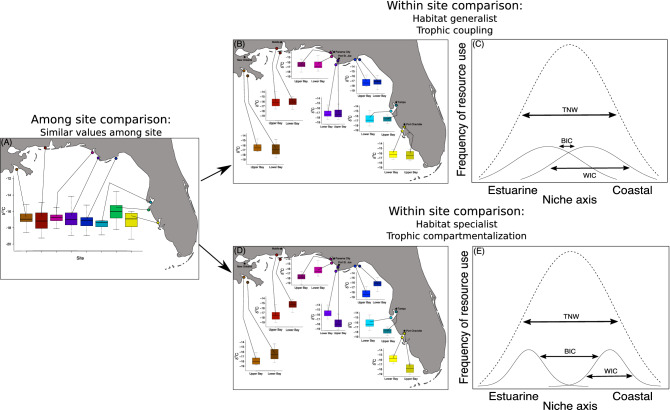
Hypothetical model depicting when isotopic values among sites are similar (**A**) and two scenarios of corresponding within-site patterns that can occur due to trophic coupling (**B**,**C**) or compartmentalization (**D**,**E**). In (**B**) dolphins sampled within and outside bays and sounds have similar, intermediate isotope values, indicating individuals use both estuarine and nearshore, marine habitats. (**C**) Individual dolphins have broad niches and the within-individual component (WIC) comprises most of the total niche width (TNW), indicating trophic coupling of habitats. In (**D**) dolphins sampled within and outside bays and sounds have different isotopic values, indicating individual dolphins use either estuarine or nearshore, marine habitats. (**E**) Individuals have narrow niches and the between-individual component (BIC) comprises most of the TNW, indicating dolphins maintain trophic compartments between habitats. Maps were generated using the ggmap package (version 3.0.0) in R^106^.

Stable isotopes are frequently used as natural tracers to delineate food webs and define trophic and habitat use or specialization^9,39,40^. Stable isotopes vary predictably among habitats and trophic levels^41,42^. For example, stable carbon isotopes are enriched in marine versus estuarine habitats, while stable nitrogen isotopes become enriched with trophic level within those habitats (i.e., browser/grazer, omnivore, primary/secondary predator)^41,43^. For dolphins that use estuarine and adjacent marine habitats, we can consider two hypothetical niche situations through which stable isotope ratios within a site could indicate trophic coupling or compartmentalization between these habitats, despite similar ratios among sites (Fig. 1A). In the first scenario (Fig. 1B), mean carbon isotope values are similar between dolphins sampled in upper and lower reaches of embayments, indicating individuals use both estuarine and marine-influenced habitats and trophically couple them. In this scenario, individual dolphins have broad niches; the within-individual component (WIC) of the total niche width (TNW; i.e., entire niche breadth of the population) is much larger than the between-individual component (BIC), and individuals act as generalists, coupling habitats (Fig. 1C). In the second scenario (Fig. 1D), mean carbon isotope values are different between dolphins from upper or lower reaches of embayments, indicating individuals use either estuarine or adjacent marine habitats but not both and do not trophically couple these habitats. In this scenario, individual dolphins have narrow niches; the BIC is much larger than the WIC, and individuals act as specialists and do not couple habitats (Fig. 1E).

Here, we combined stable isotope data from dolphins sampled across the northern and eastern Gulf of Mexico (GoM; Table S1) to determine if dolphins effectively couple estuarine and marine habitats or maintain trophic compartments between these habitats. We used data from live captured dolphins across the northern and eastern Gulf of Mexico to compare isotope values among and within sites. To better understand individual habitat use and how individual habitat use may contribute to the within site isotopic patterns, we used data from multiple tissues of stranded dolphins at one site. Data were analyzed from live dolphins sampled during health assessments and/or remote biopsy in Barataria Bay, Louisiana; east Mississippi Sound, Mississippi and Alabama; and St. Andrew Bay, St. Joseph Bay, St. George Sound, Tampa Bay, Sarasota Bay, and Charlotte Harbor, Florida. These sites span ~ 1300 km of coastline. We hypothesized that dolphins from more marine habitats would have distinctly enriched δ^13^C values compared to dolphins in estuarine habitats^38,41,43^ and that δ^15^N values would vary more with trophic level than salinity among sites^42,43^. Additionally, we tested for evidence of trophic compartmentalization or coupling and individual specialization by determining the WIC and BIC of dolphins that stranded in coastal Alabama. We analyzed isotopes from multiple tissues (liver, skin, muscle), which differ in their isotopic retention times, to provide habitat information across different temporal scales and determine if individual dolphins shifted between estuarine and marine habitats. This across and within region analysis provides the first robust evidence of trophic compartmentalization in apex predators across a region, with global implications for food web stability across habitats.

## Methods

### Study sites

We collected common bottlenose dolphin samples from nine sites along the northern and eastern Gulf of Mexico (GoM) coast (Table 1): Barataria Bay, LA; east Mississippi Sound, MS and AL; and St. Andrew Bay, St. Joseph Bay, St. George Sound, Tampa Bay, Sarasota Bay, and Charlotte Harbor, Florida. These sites contain a variety of habitat types that include saltmarshes (*Spartina alterniflora*), seagrass meadows, mangroves, and sandy or muddy, non-vegetated bottoms (Table 1). Salinity patterns vary greatly among sites (Table 1). In general, sites within bays containing large freshwater inputs have up-bay to down-bay salinity gradients where habitats in upper reaches of the bay are more freshwater-influenced than habitats in the lower reaches of the bays. For example, Barataria Bay, which is adjacent to the Mississippi River Delta, has a strong freshwater influence, whereas St. Andrew Bay, St. Joseph Bay, and the areas where St. George Sound dolphins were sampled have relatively little freshwater influence (Table 1). Sounds and sites that are open to the GoM generally have higher salinities and are less freshwater-influenced, except Mississippi Sound, which is adjacent to Lake Pontchartrain to the west and Mobile Bay to the east, both of which are large freshwater drainage basins and has several large rivers that drain directly into it (i.e., Pearl, Wolf, and Pascagoula Rivers).

**Table 1:** Attributes of each sampling site, including size (embayment/water area), type (bays are enclosed, sounds are semi-enclosed, and Gulf sites are not enclosed), salinity and freshwater influence (annual average cubic meters per second for all major tributaries), habitat types, maximum depth, and citations for habitat information.

Site	Size km^2^	Type	Salinity (ppt)	Mean freshwater discharge (m^3^s^−1^; ± SD)	Habitats	Max. depth (m)	Citations
Barataria Bay (BAR)	1116	Bay	0–30	15.3 (11.3)	Oyster beds, salt marshes, muddy bottoms	4	^95,96^
Eastern MississippiSound (MSS)	2129	Sound	4–> 35	928.5 (445.5)	Oyster beds, salt marshes, seagrasses, muddy bottoms	6	^97,98^
St. Andrew Bay (SAB)	277	Bay, Sound, Open	20–> 35	15.7 (5.6)	Urbanized, seagrasses, muddy/sandy bottoms	3	^71,99^
St. Joseph Bay (SJB)	233	Bay, Open	> 35	Negligible	Seagrasses, muddy/sandy bottoms	9	^71,100^
St. George Sound (SGS)	623	Sound, Open	15–> 35	15.3 (11.3)	Seagrasses, muddy/sandy bottoms	10	^101,102^
Tampa Bay (TMB)	1039	Bay, Open	9–33	24.2 (11.6)	Urbanized, seagrasses, mangroves, muddy/sandy bottoms	10	^103,104^
Sarasota Bay (SAR)	135	Bay		Negligible	Urbanized, seagrasses, mangroves, sandy bottoms	4	^36^
Charlotte Harbor (CLH)	700	Bay, Open	1–> 35	65.4 (23.6)	Urbanized, seagrasses, mangroves, sandy bottoms	6.1	^75,105^

### Dolphin tissue sampling

Dolphin skin samples were collected in Barataria Bay (2011, 2013, and 2018), east Mississippi Sound (2013 and 2018), and Sarasota Bay (2018) during brief capture-release for health assessment field studies. Capture-release methodologies for small cetacean health assessments have been previously detailed^44,45^, and some of these SI data have been described elsewhere^46^. Dolphin skin samples were collected in St. Andrew Bay (2004 and 2005), St. Joseph Bay (2004–2006 and 2008), St. George Sound (2004), Tampa Bay (2004), Sarasota Bay (2004, 2005, and 2008), and Charlotte Harbor (2004) during remote biopsy surveys as previously detailed^31,47,48^. Skin samples from health assessments and remote biopsy surveys for isotope analysis were collected either from the flank of the animal below the dorsal fin and above the midline or from the dorsal fin. The protocols for health assessments and remote biopsy surveys were designed with dolphin welfare and team safety being the utmost considerations to ensure all animals were sampled safely by the research team. In addition to collecting skin samples from live-captured dolphins, we obtained liver, skin, and muscle samples from five dolphins that stranded coastal Alabama waters, including east Mississippi Sound, Mobile Bay, Gulf Shores, and Orange Beach, AL in each of the following years: 2011, 2015, 2017, and 2018 (N = 20 dolphins). These tissues incorporate and retain isotopes at different rates and thereby provide information on habitat use from different time periods. Skin has a half-life of approximately 3–5 weeks^49^, and while the half-lives of liver and muscle are unknown in dolphins, the general pattern across species is that liver has half-life of 2–3 weeks and muscle 5–8 weeks^50,51^. By comparing these tissues, we are examining short, mid, and longer-term habitat use within individual dolphins^49–51^. All tissues from both live and stranded dolphins were collected in accordance with relevant guidelines and regulations, and our methodology followed the ARRIVE recommendations.

### Stable isotope analysis

We used stable isotope ratios in skin samples to define habitat use. Details on sample preparation are provided in^28,31,46,52^, and some data were previously reported in^28,31,46,52^. Samples from 2004 to 2006 and 2008 were freeze-dried to constant weight and then ground to a homogenized powder using a stainless-steel roller ball mill. Samples were analyzed on a Delta isotope ratio mass spectrometer at the FSU National High Magnetic Field Laboratory (Tallahassee, FL). Samples from 2011 and 2013 were freeze-dried up to 24 h and lipid extracted using a 2:1 chloroform:methanol mixture for 48 h in a Soxhlet extractor^46^. The lipid-extracted samples were analyzed at IsoForensics (Salt Lake City, UT) and the Stable Isotope Ratio Facility for Environmental Research (University of Utah). Skin, liver, and muscle samples from dolphins that stranded in coastal Alabama waters during 2011, 2015, 2017, and 2018, were stored at – 20 °C until processing, at which point all samples were rinsed with ultra-pure (UP) water and lipid extracted using a modified Folch method^53,54^. The lipid-extracted samples were analyzed at the Stable Isotope Facility of University of California at Davis.

All samples were weighed to ~ 1 mg and packed into tin capsules. Isotopic values were expressed using delta notation (δ) in parts per thousand (‰), where δX = (R_sample_/R_standard_ – 1) × 1000, with R_sample_ and R_standard_ representing the molar ratios of C^13^/C^12^ and N^15^/N^14^ of the sample and standard reference material, respectively. The reference material was Vienna-Pee Dee belemnite for carbon and atmospheric N_2_ for nitrogen. Analytical precisions for 2004–2006 and 2008 isotope analyses were determined from multiple measures of a single tissue analyzed in all sample runs. Based on standard deviations of these measurements (n = 8) analytical precision for δ^13^C was 0.2‰ and for δ^15^N the analytical precision was 0.4‰. Repeated analysis of in-house reference materials for the 2011 and 2013 samples were 0.02‰ for C and 0.04‰ for N and for the 2018 samples were 0.08‰ for C and 0.07‰ for N.

### Statistical analysis

For the samples from live dolphins, we used one-factor analysis of variance (ANOVA) to determine differences for each isotope type (δ^13^C and δ^15^N) among all sites across the GoM. We divided sites into upper, mid, lower subsites depending on whether dolphins were captured or biopsied in the upper parts of embayments, middle of embayments (St. Joseph Bay and St. George Sound), and lower parts of embayments or just outside of them. Depending on the number of subsites within each site, we used either ANOVAs or Welch’s t-tests to test for within-site differences: ANOVAs when there were more than two sub-sites and t-tests when there were two sub-sites. In SAR, we did not conduct a within site analysis because dolphins were sampled evenly across the site and not in discrete locations such as they were at the other sites. We tested the normality of the residuals graphically.

For the samples from stranded dolphins, we used 95% ellipses from the *SIBER* package^55^ to estimate the isotopic niche widths of individuals and all the dolphins by year and as a whole. We measured the isotopic niche space of each stranded dolphin using three tissues: liver, skin, and muscle. These ellipses are analogous to 95% confidence intervals in two-dimensions (δ^13^C and δ^15^N). We estimated the 95% ellipses of the total niche width (TNW) for each year and measured the difference in size of ellipses among years and the proportion of overlap among ellipses of different years. While the size of the ellipses varied among years, with 2011 having the smallest and 2017 the largest (Tables S1, S2 and Figure S1), there was considerable overlap in ellipses among years (Table S3 and Figure S1). As such, we combined all individuals into a single ellipse that represented the TNW for all individuals and all years (Table S1). Our isotopic approach to measuring the within-individual component (WIC) and between-individual component (BIC) of the total niche width (TNW) is based on Roughgarden (1972), where the TNW = WIC + BIC. We divided the ellipse of each individual by the TINW to obtain the within-individual component (WIC) and the between-individual component (BIC) = 1 − WIC^56,57^. For example, if the ellipse for the entire population (TINW) = 15‰^2^ and the averaged individual ellipses is 3‰^2^, than the WIC = 3/15 = 0.33 and the BIC = 0.67. In this case, the WIC in much smaller component of the TNW than the BIC and individuals are specialists when the population as a whole is generalist. Therefore, WIC/TNW is a measure of individual specialization where values closer to 0 indicate individual specialists while those closer to 1 indicate individual generalists. We fitted all the δ^13^C and δ^15^N values to the normal distribution and calculated the average δ^13^C and δ^15^N values. All statistical analyses were performed in R^58^.

## Results

### Among-site comparisons of live dolphins

**Figure 2:**
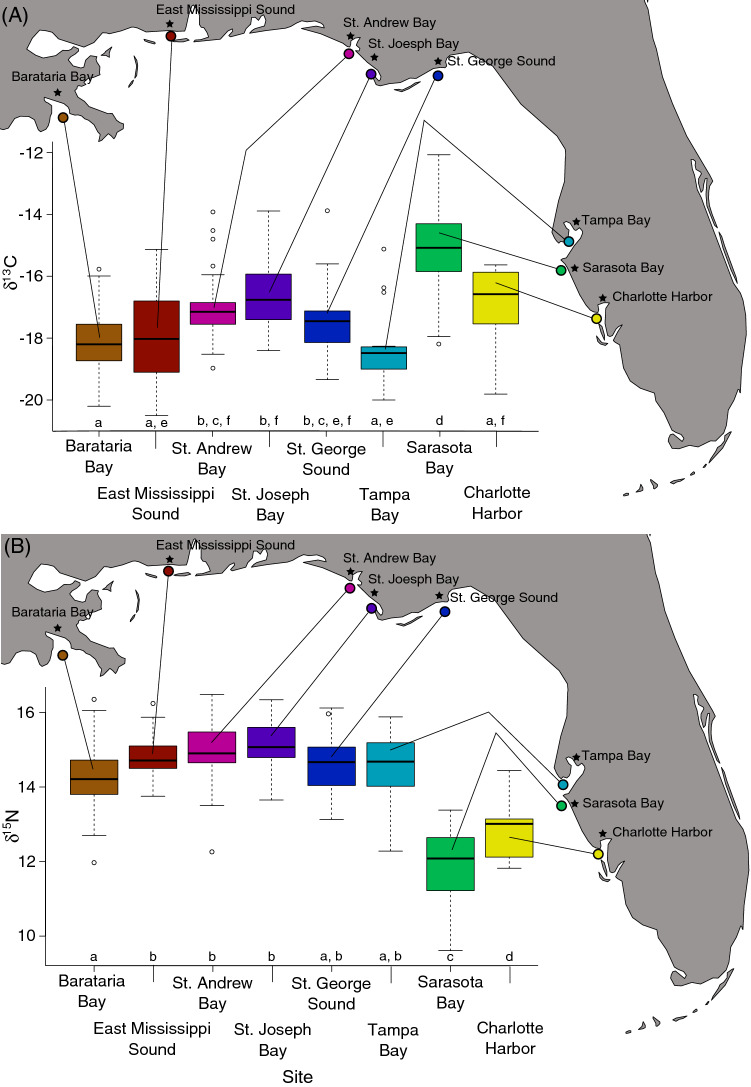
Among site variation in δ^13^C (**A**) and δ^15^N (**B**) values. Letters below boxplots indicate which sites are statistically similar*.* Maps were generated using the ggmap package (version 3.0.0) in R^106^.

The δ^13^C values in dolphin skin ranged from − 20.50 to − 12.07‰ and differed among some of the sites (Fig. 2A; Table 1; F_7, 301_ = 44.4, *p* < 0.001). Sarasota Bay had enriched δ^13^C values compared to the other sites, including the adjacent sites of Tampa Bay and Charlotte Harbor (Fig. 2A; Table 2), while other sites had similar δ^13^C values despite considerable distance between them (Fig. 2A; Table 2). For example, Barataria Bay had δ^13^C values similar to Charlotte Harbor and Tampa Bay (Fig. 2A; Table 2).

**Table 2:** Mean δ^13^C and δ^15^N values (± 95% CI) and sample sizes (N = number of dolphins) from each site in the nGoM and sampling locations within each site.

Site (N)	Sample location (N)	δ^13^C ‰ (± 95% CI)	δ^15^N ‰ (± 95% CI)
Barataria Bay (113)		− 18.17 (0.14)	14.26 (0.12)
	Upper bay (10)	− 18.72 (0.07)	13.98 (0.08)
	Lower bay (41)	− 17.91 (0.13)	14.50 (0.09)
Mississippi Sound (34)		− 18.00 (0.39)	14.81 (0.15)
	Upper: estuary (15)	− 19.19 (0.27)	14.85 (0.13)
	Lower: island (17)	− 16.99 (0.24)	14.76 (0.18)
St. Andrew Bay (27)		− 16.93 (0.36)	14.94 (0.30)
	Upper: bay (14)	− 17.53 (0.22)	14.47 (0.27)
	Lower: sound (13)	− 16.29 (0.38)	15.45 (0.26)
St. Joseph Bay (22)		− 16.61 (0.40)	15.16 (0.23)
	Upper: St. Joseph Bay (5)	− 16.08 (0.54)	14.92 (0.22)
	Mid: Mexico Beach (10)	− 16.59 (0.24)	15.23 (0.23)
	Lower: Gulf side-St. Joseph Bay (7)	− 17.40 (0.32)	15.38 (0.24)
St. George Sound (28)		− 17.36 (0.36)	14.72 (0.28)
	Upper: St. George Sound (9)	− 16.90 (0.47)	15.54 (0.28)
	Mid: Turkey Point (5)	− 17.28 (0.32)	14.70 (0.16)
	Lower: Alligator Point (14)	− 17.70 (0.26)	14.21 (0.19)
Tampa Bay (15)		− 17.75 (0.66)	14.12 (0.24)
	Upper: Inner Bays (8)	− 17.75 (0.66)	14.12 (0.24)
	Lower: Lower Tampa Bay (7)	− 18.83 (0.15)	15.03 (0.47)
Sarasota Bay (59)		− 15.05 (0.31)	11.89 (0.21)
Charlotte Harbor (9)		− 17.04 (0.71)	12.84 (0.43)
	Upper bay (2)	− 19.18 (–)	13.73 (–)
	Lower lower (7)	− 16.42 (0.40)	12.58 (0.31)

The δ^15^N values in dolphin skin ranged from 9.61‰ to 17.12‰ and also differed among some sites (Fig. 2B; F_7, 302_ = 79.96, *p* < 0.001). Dolphins at most sites along the northern GoM had similar δ^15^N values that were enriched compared to the easternmost sites of the GoM (Sarasota and Charlotte Harbor), which also differed from each other (Fig. 2B; Table 2).

### Within-site comparisons of live dolphins

**Figure 3:**
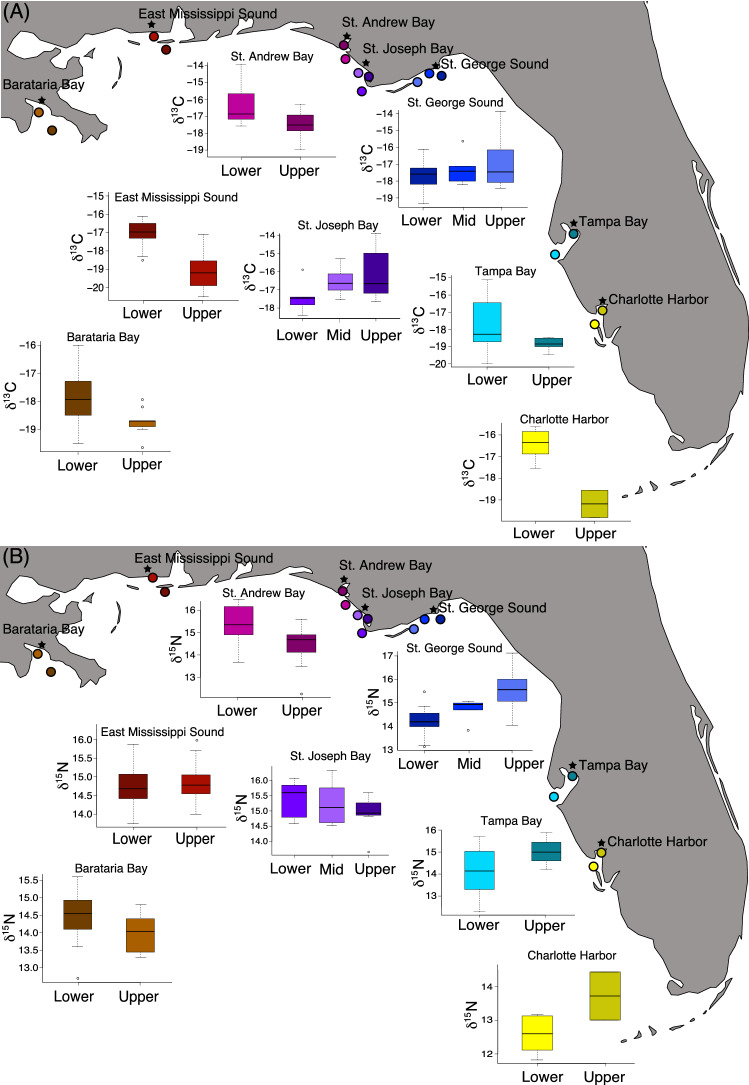
Within﻿ site variation in δ^13^C (**A**) and δ^15^N (**B**) values. Maps were generated using the ggmap package (version 3.0.0) in R^106^.

Generally, the δ^13^C values in dolphin skin differed considerably within sites, ranging up to 5.35‰ at East Mississippi Sound for example (Fig. 3A; Tables 2 and 3). These differences were greatest at sites with strong estuarine to marine gradients that had large freshwater supplies, such as Barataria Bay, Mississippi Sound, St. Andrew Bay, Tampa Bay, and Charlotte Harbor (Table 1). In these locations, dolphins sampled in lower reaches and outside of embayments had enriched δ^13^C values compared to dolphins sampled in upper reaches of embayments (Fig. 3A; Tables 2 and 3). In Tampa Bay, dolphins sampled in the upper parts of the bay had restricted and depleted δ^13^C values compared to lower reaches of the bay, in which the full range of values was observed even though most δ^13^C values were comparatively enriched (Fig. 3A; Tables 2 and 3). In Charlotte Harbor, only two dolphins were sampled from upper reaches; consequentially, we did not make statistical comparisons to stable isotope values in these dolphins. The δ^13^C values were similar within sites lacking clear estuarine and marine habitat boundaries, such as St. Joseph Bay, or where most dolphins were sampled distant from major freshwater input, such as St. George Sound (Fig. 3A).

**Table 3:** Welch’s t test and ANOVA statistics for within site comparisons.

Isotope	Site	df	Test statistic	*p*
δ^13^C	Barataria Bay	25.22	− 4.23	< 0.001
	Mississippi Sound	28.15	− 7.04	< 0.001
	St. Andrew Bay	19.07	− 3.24	0.004
	St. Joseph Bay	2, 19	2.23	0.135
	St. George Sound	2, 25	1.39	0.268
	Tampa Bay	7.82	− 1.85	0.103
δ^15^N	Barataria Bay	15.63	− 2.76	0.014
	Mississippi Sound	28.38	0.43	0.669
	St. Andrew Bay	24.99	− 3.03	0.006
	St. Joseph Bay	2, 19	0.81	0.460
	St. George Sound	2, 25	9.77	< 0.001
	Tampa Bay	10.48	1.98	0.074

T tests were performed when there were two locations per site and ANOVAs when there were more than two locations per site.

The δ^15^N values in dolphin skin also differed within some sites, ranging up to 3.99‰ at Charlotte Harbor (Fig. 3B; Tables 2 and 3). Dolphins sampled from the upper reaches of bays typically had enriched δ^15^N values compared to those in the lower bays (Fig. 3B; Table 2). However, in Barataria Bay, dolphins sampled lower in the bay had enriched values compared to those sampled in the upper bay (Fig. 3B; Tables 2 and 3). The δ^15^N values also differed within the more open water sites at St. Andrew Bay and St. George Sound (Fig. 3B; Tables 2 and 3).

### Niche width components of dolphins

**Figure 4:**
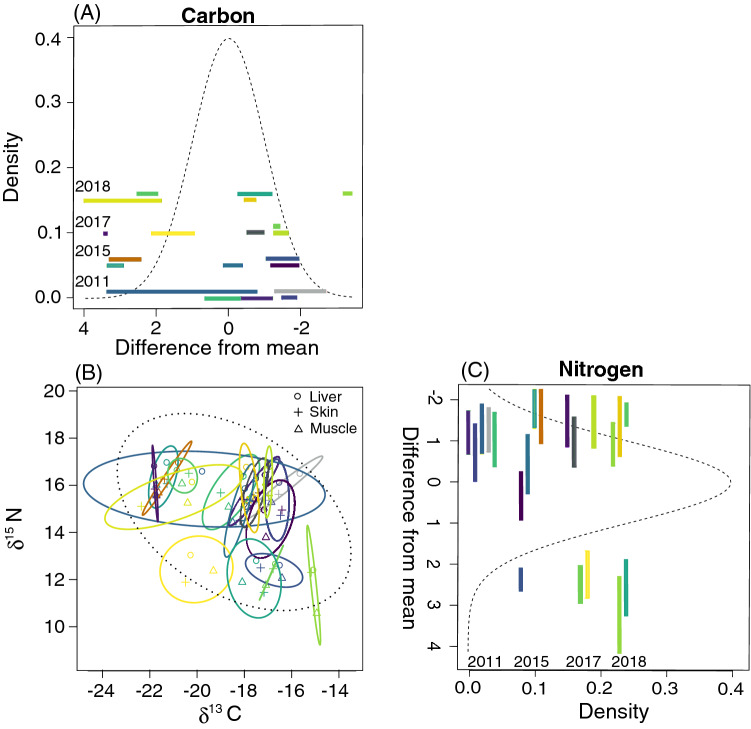
Individual variation in δ^13^C (**A**) and δ^15^N (**C**) values of stranded dolphins from Alabama. Ellipses (**B**) represent the 95% confidence intervals of δ^13^C and δ^15^N values in each individual calculated using three tissue types (liver, skin, muscle). Each color corresponds to an individual, with ellipse size corresponding to isotopic niche size. The black, dotted ellipse reflects the niche size for all tissues from all individuals. The normal distributions of our δ^13^C and δ^15^N values are presented as the dashed lines in (**A**) and (**B**), respectively. For (**A**), the panel has been presented as the mirror image of the original because the negative values associated with δ^13^C flip the signs of the differences (i.e., − to + and + to −) when subtracted from the mean, which explains why the x-axis goes from positive to negative. The colored bars in (**A**) and (**B**) indicate the range of differences between the mean δ^13^C (− 18.4‰) or δ^15^N (14.9‰) value and each tissue for every individual. Individuals were organized by year along the y-axis to help space the colored bars and prevent them for overlapping and are not related to the values on the y-axis.

Similarities of δ^13^C values among tissues within individuals suggested localized habitat use and narrow niche width of stranded dolphins from coastal Alabama waters. Since these dolphins stranded dead, we do not know their spatial use within coastal Alabama waters prior to stranding and could not categorize them as using upper or lower parts of the bay. However, there were clear differences in δ^13^C values among individuals that are likely associated with habitat use and little variation among tissue types within each individual (Fig. 4A,B). In Fig. 4B, each solid ellipse represents the δ^13^C and δ^15^N isotopic niche space of one individual measured from the three tissues, and the black dashed line indicates the total isotopic niche width (TINW) of all stranded dolphins combined (N = 20). Most solid ellipses are small, and there is little isotopic variation within individuals, suggesting that individuals remained in similar habitats in the weeks-to-months prior to stranding. When solid ellipses are large (blue ellipse from 2011; Fig. 4A,B), there is greater isotopic variation within individuals. The averaged ellipse area across individuals was 0.602‰^2^, while the area for all individuals was 9.758‰^2^, and the averaged WIC (0.062) was smaller than the averaged BIC (0.938) (Table S1). Most of the variation in TINW was between individuals, consistent with most individuals being habitat specialists (Fig. 4; Table S1). We also found considerable differences between individuals when comparing the distribution of δ^13^C values to the mean of all samples (− 18.4‰; Fig. 4B). For example, an individual sampled in 2017 (Fig. 4B, bright yellow) had a range of differences from the carbon global mean between − 2‰ and − 1‰, which is a small range compared to all dolphins. These carbon differences suggest that most individuals, except the 2011 individual (Fig. 4B, long blue line), were using the same habitats in the weeks-to-months prior to stranding.

For δ^15^N values, which provide information on trophic level, there was greater variation within individuals compared to δ^13^C values (i.e., colored bars are longer; Fig. 4C). Most individuals had similar δ^15^N values and a similar range of differences from the global nitrogen mean (14.9‰; Fig. 4C). Five individuals that stranded in Orange Beach, Alabama had depleted δ^15^N values compared to those that stranded in Mobile Bay or on Dauphin Island, Alabama, representing the greatest differences in δ^15^N values from the global mean.

## Discussion

Our results suggest that individual habitat specialization of northern GoM dolphins limits trophic coupling and may contribute to widespread trophic compartmentalizing within energetically distinct estuarine and adjacent, nearshore marine habitats. Dolphins sampled in upper parts of embayments at most sites had depleted δ^13^C values indicative of estuarine habitats, while dolphins sampled in lower parts of embayments had enriched δ^13^C values indicative of nearshore, marine habitats. Only two dolphins were sampled in the upper reaches of Charlotte Harbor, but the differences in δ^13^C values between these animals and those from lower sections of the harbor were similar to those found in other estuaries throughout the GoM. These findings are consistent with the expected pattern of dissolved inorganic carbon sources in freshwater influenced estuarine systems versus marine areas^41,43^. Furthermore, analysis of multiple tissues from the same individuals from coastal Alabama revealed little within-individual variation and considerable between-individual variation in δ^13^C values. The within individual analysis revealed that most individuals have either enriched or depleted δ^13^C values across tissues, suggesting individuals are consistently using either lower or upper sections of Mobile Bay and surrounding waters, similar to dolphins at other sites with a strong estuarine-marine gradient^41,43^. These results provide evidence that northern GoM dolphins use a narrow range of available habitats and are individual habitat specialists, rarely using and foraging in both estuarine and nearshore marine habitats. Our isotopic results are similar to telemetry-based and observational movement data that have demonstrated discontinuous habitat use between dolphins in estuarine and nearshore, marine habitats worldwide^26,27,59,60^.

Habitat specialization may be globally common among estuarine dolphin populations. In general, dolphins are capable of extended movements that allow them to use multiple habitats^28,61,62^. For example, many populations of nearshore and offshore dolphins have ranges that are 100’s to 1000’s of km^2^^24,63^. In contrast, numerous telemetry and photo-identification studies have shown that estuarine dolphins frequently exhibit high site fidelity and localized movements ^27,33,64^. Estuarine dolphins in this study and others have ranges between 1 and ~ 500 km^2^, with most individual ranges $<$ 200 km^2^^26,32,63^. The limited movements of estuarine dolphins may be driven at least in part by the high abundance of resources found in these habitats^65,66^ such that estuarine dolphins may only require relatively small ranging patterns to meet their nutritional requirements. In nearshore and offshore habitats, where prey are more patchily distributed, dolphins likely need larger ranges to forage^24,67^. Social factors may also facilitate constrained ranges, which remain consistent among generations as prey capture techniques are learned and often specific to particular prey at certain sites^21,29,35^. Our results suggest that restricted habitat use by estuarine dolphins in the GoM leads to habitat specialization and subsequent compartmentalization of estuarine and adjacent, nearshore marine habitat use that is regionally widespread. Likewise, similar movement patterns occur in several Brazilian estuaries and a Scottish bay^33,34,64^. Given the similar movement patterns of estuarine dolphins worldwide, this finding may reflect a global pattern of limited trophic, and thus habitat, coupling between estuarine and adjacent marine habitats by dolphins.

The overall pattern of δ^13^C values among sites across the northern and eastern GoM are generally predictable based on the ecological setting. The variation in δ^13^C values among sites is likely driven by differences in environmental factors such as freshwater input, nutrient inputs, and seagrass coverage^41^. Sites with high freshwater input, such as Barataria Bay, Mississippi Sound, and Tampa Bay, had depleted δ^13^C values, as would be expected from the associated terrestrial contributions to these systems^20,41^. Sites without high freshwater input and the associated estuarine-marine habitat gradient had similar δ^13^C values throughout the sites^68,69^. Sarasota Bay has extensive seagrass beds with minimal freshwater inputs, and many resident dolphins that we sampled likely feed upon seagrass-associated fish, as they were captured or sampled in shallow-water habitats within the bay^20^. Thus, it is not surprising that dolphins from Sarasota Bay had isotopic signatures similar to other systems dominated by seagrasses i.e., enriched in carbon isotopes and depleted in nitrogen isotopes^70^. The upper reaches of St. Joseph Bay and St. George sound had the most enriched δ^13^C values, opposite sites with a strong estuarine-marine gradient. Because of their lack of freshwater inflow, the upper reaches of these sites have the greatest concentration of seagrasses at those sites and the most enriched δ^13^C values^28,71^, and dolphins at the remaining areas of these sites do not appear to feed as much on seagrass-associated fish compared to Sarasota Bay^28^. Tampa Bay also has extensive seagrass beds but also has freshwater input and considerable nutrient input from the surrounding urban landscape that can contribute to depleted δ^13^C values in the system^72,73^. St. Andrew Bay and Charlotte Harbor both have seagrass beds^74,75^ and freshwater inputs, resulting in δ^13^C values intermediate between the seagrass-dominated Sarasota Bay and other freshwater-influenced sites like Barataria Bay, Mississippi Sound, and Tampa Bay.

The relatively similar δ^15^N values of dolphins among sites suggests that dolphins are feeding at similar trophic levels globally^28,76,77^. The δ^15^N values were relatively high for coastal GoM, corroborating other research that dolphins feed at top trophic levels in these systems^20,28^. The sites with the most different δ^15^N values included Sarasota Bay and Charlotte Harbor, and the depleted δ^15^N values in dolphins at these sites are likely caused by feeding on seagrass-associated fish, which tend to have lower δ^15^N values^20,28^. Alternatively, higher rates of N-fixation may result in low δ^15^N values in Sarasota Bay and Charlotte Harbor. *Trichodesmium* are N-fixing cyanobacteria in the GoM, and *Trichodesmium* blooms have historically been more frequent near Sarasota Bay and Charlotte Harbor than at other GoM sites^78,79^. These patterns suggest that dolphins are feeding on relatively similar prey at the same trophic level across the GoM, with some minor site-driven differences^20,28,80^.

Our results challenge the notion that trophic coupling is widespread and common in coastal habitats^2,8,11,12^. Although dolphins are highly mobile and are capable of moving among habitats and trophically coupling them, previous studies have demonstrated that estuarine dolphins do not necessarily leave their localized ranges even during extreme disturbances. For example, during the *Deepwater Horizon* oil spill, dolphins remained in Barataria Bay and, as a result, suffered severe health impacts with individual and population level effects^81–84^. Likewise, at both Barataria Bay and Mississippi Sound, dolphins remained within their localized ranges during periodic incursions of freshwater that caused poor health and mortality, including incursions during unusual mortality events^85,86^. Similarly, Sarasota Bay dolphins have altered their diet but not their ranging pattern during harmful algae blooms in which prey abundance and distribution drastically changed^87^. Dolphins have dietary flexibility that may help them resist ecological changes within their localized ranges^28,88^, but evidence increasingly indicates that dolphins are unlikely to leave localized ranges even when associated with adverse impacts^26,32,81,82,89^. Dolphins not leaving during periods of extreme stress is counter to preconceived notions on dolphins and other species with apparent habitat plasticity, and we encourage researchers to explore these unexpected patterns and not ignore them. Similar work in Florida demonstrates that another mobile, apex predator, the bull shark (*Carcharhinus leucas*), also does not couple estuarine and marine habitats as previously thought^9^. Thus, these food webs could be less stable than previously thought^7,90^, and this lack of stability could make habitats like estuaries more prone to ecosystem changes and disturbances if food web perturbations are not dampened by trophic coupling from adjacent nearshore, marine habitats.

The lack of habitat coupling may make these systems prone to food web perturbations, particularly because other common stressors in these habitats (e.g., harmful algal blooms, freshwater discharge and salinity variation, shipping and vessel traffic, oil spills, climate disruption) can cause adverse population-level effects on dolphins^81,91^ that may, in turn, have serious cumulative, down-food web consequences not dampened by trophic coupling^7,90^. Estuarine habitats pose challenges for bottlenose dolphins and some sharks because of the high variation in salinity levels, and large, teleost fish and other shark species may be better suited and more important to stabilizing estuarine food webs than dolphins. More food web studies across habitats are required to quantify the extent, if any, of coupling by dolphins or other species. Current research suggests that many fish that use both estuarine and marine habitats vary habitat use with ontogeny, using estuaries as nurseries and subsequently leaving the estuary after a period of growth, only to return for breeding purposes^92,93^. As such, not all large teleost fish may functionally couple estuarine and adjacent marine habitats^94^. Additional work is needed to investigate the extent of trophic coupling among species resident in coastal systems, the contribution of dolphins and other apex predators to coupling or maintaining trophic compartments, and the potential for complex interactions between coupling and compartmentalization by different species to affect pulse and longer-term ecosystem stability.

## Supplementary Information

Supplementary Information.

## Data Availability

Data are publicly available through the Gulf of Mexico Research Initiative Information & Data Cooperative (GRIIDC) at https://data.gulfresearchinitiative.org (stable isotope values https://doi.org/10.7266/n7-e5ry-ck72), except for data collected in Barataria Bay, LA and Mississippi Sound, MS in 2013, which can be found under the Deepwater Horizon Natural Resource Damage Assessment Data in the DIVER database, https://www.diver.orr.noaa.gov.
