# Supplementary material for: Specialization of a mobile, apex predator affects trophic coupling among adjacent habitats
Source: Sci Rep. 2021 Oct 4;11:19611. doi: 10.1038/s41598-021-99017-1 (PMC8490471; doi:10.1038/s41598-021-99017-1)
Supplement: Supplementary file 1 — Supplementary Information. [file 41598_2021_99017_MOESM1_ESM.pdf]

Supplemental Information for

Specialization of a mobile, apex predator affects trophic coupling among adjacent habitats

Carl S. Cloyed, Rachel M. Wilson, Brian C. Balmer, Aleta A. Hohn, Lori H. Schwacke, Eric S. Zolman, Mandy C. Tumlin, Randall S. Wells, Aaron A. Barleycorn, Jason B. Allen, Ruth H. Carmichael

This file contains:

Table S1: Attributes of each sampling site.

Table S2: Standard Bayesian ellipses corrected for small sample size.

Table S3: *p*-values for pairwise differences in standard Bayesian CN ellipses areas corrected for small sample size (SEAc) among years for dolphin skin from Mobile Bay, AL.

Table S4: Pair-wise proportional overlap in standard Bayesian CN ellipses corrected for small sample size (SBEc) among years of dolphin skin from Mobile Bay, AL.

Figure S1: Standard Bayesian Ellipses corrected for small sample sizes (which represent multivariate 95% credible intervals of the of $\delta^{13}C$ and $\delta^{15}N$ values) for each year.

Table 1: Attributes of each sampling site, including size (embayment/water area), type (bays are enclosed, sounds are semi-enclosed, and Gulf sites are not enclosed), salinity and freshwater influence (annual average cubic meters per second for all major tributaries), habitat types, maximum depth, and citations for habitat information.

Site	Size km^2	Type	Salinity (ppt)	Mean freshwater discharge (m^3s^{-1}; ± SD)	Habitats	Max. Depth (m)	Citations
Barataria Bay (BAR)	1,116	Bay	0 - 30	15.3 (11.3)	Oyster beds, Salt marshes, Muddy bottoms	4	(1, 2)
Eastern Mississippi Sound (MSS)	2,129	Sound	4 - > 35	928.5 (445.5)	Oyster beds, Salt marshes, Seagrasses, Muddy bottoms	6	(3, 4)
St. Andrew Bay (SAB)	277	Bay, Sound, Open	20 - > 35	15.7 (5.6)	Urbanized, Seagrasses, Muddy/sandy bottoms	3	(5, 6)
St. Joseph Bay (SJB)	233	Bay, Open	> 35	Negligible	Seagrasses, Muddy/sandy bottoms	9	(5, 7)
St. George Sound (SGS)	623	Sound, Open	15 - > 35	15.3 (11.3)	Seagrasses, Muddy/sandy bottoms	10	(8, 9)
Tampa Bay (TMB)	1,039	Bay, Open	9 – 33	24.2 (11.6)	Urbanized, Seagrasses, Mangroves, Muddy/sandy bottoms	10	(10, 11)
Sarasota Bay (SAR)	135	Bay		Negligible	Urbanized, Seagrasses, Mangroves, Sandy bottoms	4	(12)
Charlotte Harbor (CLH)	700	Bay, Open	1 - > 35	65.4 (23.6)	Urbanized, Seagrasses, Mangroves, Sandy bottoms	6.1	(13, 14)

Table S2: Standard Bayesian ellipses corrected for small sample size (SBE_c) and the estimate lower and upper 95% credible intervals for each stranded dolphin, as well as for all individuals in all years, and for all individuals within each year. Larger SBE_c's indicate larger isotopic niches. Within-individual components (WIC) and between-individual components (BIC) were measured for both all individuals and all years (WIC All Years = $SBE_i/SBE_{All, All}$, where SBE_i is the SBE_c of the *i*th individual and $SBE_{All, All}$ is the SBE_c from all individuals from all years and BIC All Years = 1 – WIC All Years) and for all individuals within years (WIC Within Year = $SBE_i/SBE_{All, j}$, where SBE_i is the SBE_c of the *i*th individual and $SBE_{All, j}$ is the SBE_c from all individuals in year *j* and BIC Within Year = 1 – WIC Within Year).

Field ID	SBE_c	Lower 95% CI	Upper 95% CI	95 CI	WIC All Years	BIC All Years	WIC Within Year	BIC With Year
10DISL072211	0.629	0.146	2.856	2.71	0.064	0.936	0.237	0.763
14DISL090811	0.298	0.062	1.292	1.23	0.031	0.969	0.112	0.888
19DISL100711	2.598	0.663	12.494	11.831	0.266	0.734	0.979	0.021
23DISL101311	0.809	0.215	3.893	3.678	0.083	0.917	0.305	0.695
28DISL120911	0.795	0.181	3.312	3.131	0.081	0.919	0.300	0.700
06DISL031515	0.704	0.154	3.184	3.03	0.072	0.928	0.103	0.897
19DISL100615	0.344	0.09	1.614	1.524	0.035	0.965	0.050	0.950
20DISL111015	0.404	0.124	1.891	1.767	0.041	0.959	0.059	0.941
24DISL121815	0.301	0.069	1.506	1.437	0.031	0.969	0.044	0.956
26DISL122915	0.521	0.117	2.606	2.489	0.053	0.947	0.076	0.924
10DISL030917	0.075	0.02	0.325	0.305	0.008	0.992	0.006	0.994
13DISL032017	0.357	0.08	1.646	1.566	0.037	0.963	0.031	0.969
31DISL083117	0.17	0.048	0.832	0.784	0.017	0.983	0.015	0.985
36DISL092117	0.844	0.169	3.847	3.678	0.086	0.914	0.073	0.927
33DISL091517	0.105	0.03	0.522	0.492	0.011	0.989	0.009	0.991
23DISL061818	0.253	0.063	1.15	1.087	0.026	0.974	0.024	0.976
25DISL070718	0.65	0.156	2.895	2.739	0.067	0.933	0.061	0.939
30DISL091718	0.195	0.039	0.818	0.779	0.020	0.980	0.018	0.982
32DISL100818	0.248	0.072	1.184	1.112	0.025	0.975	0.023	0.977
34DISL102018	1.745	0.389	7.316	6.927	0.179	0.821	0.163	0.837
All Individuals, All Years	9.758	7.497	12.676	5.179	—	—	—	—
All Individuals, 2011	2.654	1.536	4.497	2.961	—	—	—	—
All Individuals, 2015	6.855	3.907	11.784	7.877	—	—	—	—
All Individuals, 2017	11.627	6.604	19.82	13.216	—	—	—	—
All Individuals, 2018	10.719	6.371	18.656	12.285	—	—	—	—

Table S3: *p*-values for pairwise differences in standard Bayesian CN ellipses areas corrected for small sample size (SEAc) among years for dolphin skin from Mobile Bay, AL.

	2011	2015	2017
2011			
2015	0.005		
2017	0.000	0.080	
2018	0.000	0.102	0.435

Table S4: Pair-wise proportional overlap in standard Bayesian CN ellipses corrected for small sample size (SBEc) among years of dolphin skin from Mobile Bay, AL. The reference population is always the year at the top of column, i.e., the very top-right value, 0.179, is the proportion of the 2018 ellipsis that overlaps with the 2011 ellipsis, whereas the bottom-left value, 0.813, is the proportion of the 2011 ellipsis that overlaps with the 2013 ellipsis.

	2011	2015	2017	2018
2011		0.219	0.224	0.179
2015	0.695		0.756	0.623
2017	0.914	0.588		0.689
2018	0.813	0.891	0.766	

Figure S1: Standard Bayesian Ellipses corrected for small sample sizes (which represent multivariate 95% credible intervals of the of $\delta^{13}C$ and $\delta^{15}N$ values) for each year.

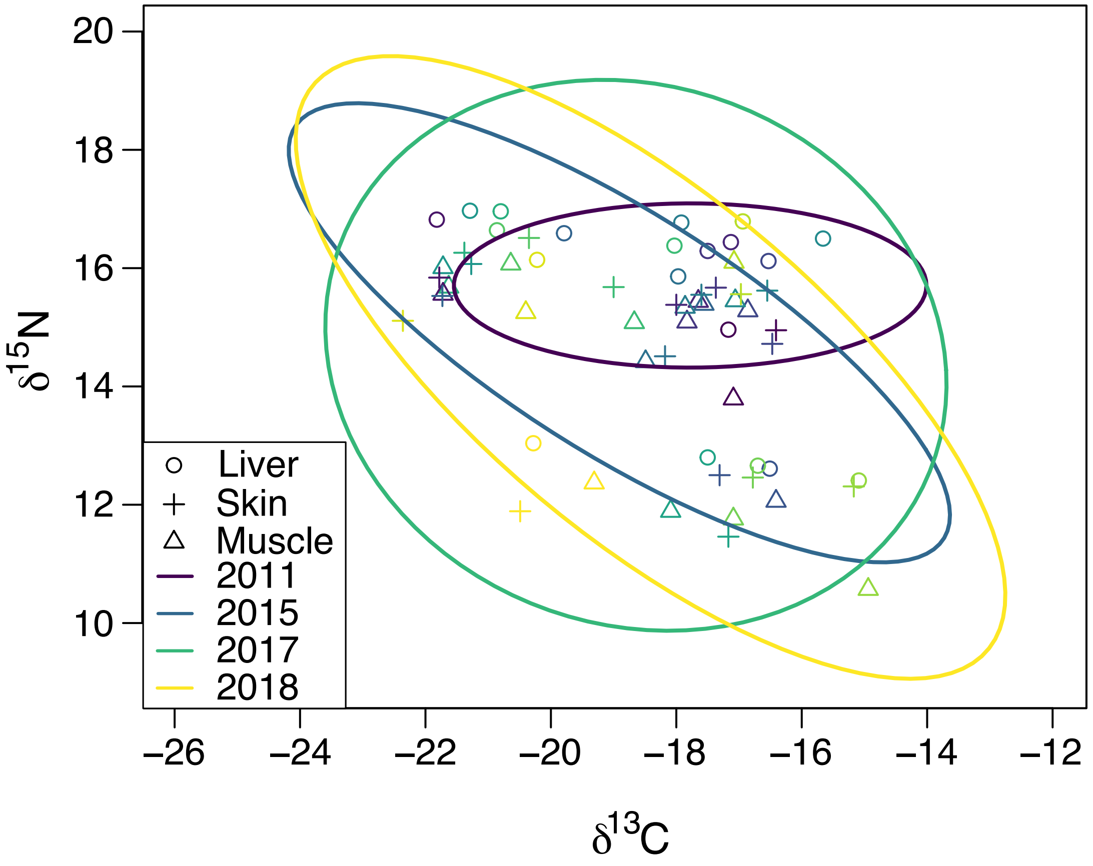

Refereneces

1. D. M. Fitzgerald, M. Kulp, S. Penland, J. Flocks, J. Kindinger, Morphologic and stratigraphic evolution of muddy ebb-tidal deltas along a subsiding coast: Barataria Bay, Mississippi River Delta. *Sedimentology* **51**, 1157-1178 (2004).
2. E. Habib *et al.*, Assessing effects of data limitations on salinity forecasting in Barataria basin, Louisiana, with a Bayesian analysis. *Journal of Coastal Research* **2007**, 749-763 (2007).
3. C. K. Eleuterius, Geographical definition of Mississippi Sound. *Gulf and Caribbean Research* **6**, 179-181 (1978).
4. K. L. Lucas, G. A. Carter, Decadal changes in habitat-type coverage on Horn Island, Mississippi, USA. *Journal of Coastal Research* **26**, 1142-1148 (2010).
5. C. A. Ward-Paige, G. L. Britten, D. M. Bethea, J. K. Carlson, Characterizing and predicting essential habitat features for juvenile coastal sharks. *Marine Ecology* **36**, 419-431 (2015).
6. T. Ichiye, M. L. Jones, ON THE HYDROGRAPHY OF THE ST. ANDREW BAY SYSTEM, FLORIDA 1. *Limnology and Oceanography* **6**, 302-311 (1961).
7. S. G. Morgan, Plasticity in reproductive timing by crabs in adjacent tidal regimes. *Marine Ecology Progress Series* **139**, 105-118 (1996).
8. R. Livingston *et al.*, Modelling oyster population response to variation in freshwater input. *Estuarine, Coastal and Shelf Science* **50**, 655-672 (2000).
9. D. Twichell *et al.*, Geologic controls on the recent evolution of oyster reefs in Apalachicola Bay and St. George Sound, Florida. *Estuarine, Coastal and Shelf Science* **88**, 385-394 (2010).
10. Z. Chen, C. Hu, R. N. Conmy, F. Muller-Karger, P. Swarzenski, Colored dissolved organic matter in Tampa Bay, Florida. *Marine Chemistry* **104**, 98-109 (2007).
11. P. Julian, E. D. Estevez (2009) Historical bathymetric analysis of Tampa Bay. in *Proceedings of the Tampa Bay Area Scientific Information Symposium, BASIS 5: Using Our Knowledge to Shape Our Future*, pp 27-33.
12. E. J. Berens McCabe, D. P. Gannon, N. B. Barros, R. S. Wells, Prey selection by resident common bottlenose dolphins (*Tursiops truncatus*) in Sarasota Bay, Florida. *Marine biology* **157**, 931-942 (2010).
13. A. J. Adams, D. A. Blewett, Spatial patterns of estuarine habitat type use and temporal patterns in abundance of juvenile permit, Trachinotus falcatus, in Charlotte Harbor, Florida. *Gulf and Caribbean Research* **16**, 129-139 (2004).
14. G. R. Poulakis, D. A. Blewett, M. E. Mitchell, The effects of season and proximity to fringing mangroves on seagrass-associated fish communities in Charlotte Harbor, Florida. *Gulf of Mexico Science* **21**, 3 (2003).
